# Medical Observations and Inquiries, Vol. VI

**Published:** 1785

**Authors:** 


III. *Medical Obfervations and Inquiries*, Vol. VI. (Continued from page 329.)

XII. *CASE of a fpafmodic Inability of Deglutition cured by mercurial Unction. By* J. H. Sequiera, *M. D.*—We have here an account of a youth, who, after having for feveral months been afflicted with an inceffant dry cough and convulfive fits, which had refifted a variety of remedies, and had impaired his mental faculties, and brought on a general tremor, refembling the St. Vitus's dance, at length perceived a difficulty of deglutition, which increafed fo faft, that, in the fpace of a few days, he became totally incapable of fwallowing any thing, either folid or liquid. On the firft appearance of this alarming fymptom opiates were adminiftered in large dofes; but thefe producing no good effect, our author was tempted to have recourfe to mercurial frictions. On the fixth day, foon after his mouth had had become affected, he began to recover the power of deglutition, which was perfectly restored a few hours afterwards. But what is most remarkable, the cough, tremor, and, in short, the whole of the complaint, vanished as if by a charm, and the patient has ever since enjoyed good health.

XIII. *The Use of Cold Bathing in the Locked Jaw*, &c. *By* William Wright, *M. D. F. R. S.* —In this paper are related several cases of the tetanus and opisthotonos, which were successfully treated by the external application of cold water. The author observes, that since he has adopted this method, (which, it seems, was first suggested to him by his friend Dr. Lind, of Haslar hospital) he has never failed, in one instance, to effect a cure; and that in a shorter time than by any other method hitherto proposed. In two of the six cases here published no mention is made of opium; in the other four, the use of this remedy was added to that of the cold water; but in all the cases the good effects of the latter were sufficiently obvious. The mode of applying it consisted in pouring two or three pailfuls over the patient's body every three or four hours; and the first application of the water in this manner generally produced produced a glowing heat, and a confiderable abatement of the fymptoms.

XIV. *Cafe of a fingular Cough. By* A. Douglas, *M. D. of London.* — The patient, whofe cafe is here related, was in her fifty-feventh year, and apparently in perfect health when fhe was attacked with this cough. She at firft felt an itching deep in the left fide of her throat, near the angle of her lower jaw, which was almoft inftantly followed by a violent fit of coughing. This fit continued for ten or twelve minutes, effort following effort in the moft rapid fucceffion, till it became neceffary to infpire; which done, the efforts returned as quick as ever. From this time the cough returned frequently, at uncertain intervals, but never twice in the fame day; and, in half an hour after the cough had ceafed, fhe was as well as if nothing had happened. A variety of medicines were adminiftered without much effect, till one day our author, who happened to be prefent when the cough feized her with uncommon violence, applied a bottle, containing ten or twelve ounces of eau de luce, to her noftrils, and at that inftant the cough ceafed. The complaint, however, returned, and continued to afflict her for feveral years; but the application application of two or more ounces of eau de luce never failed to ſtop or much ſhorten the fit, although never ſo ſuddenly or effectually as at firſt, when the bottle contained ten or twelve ounces; and, indeed, the author obſerves, that the benefit ariſing from it always appeared to be in ſome kind of proportion to the quantity contained in the phial. At length the cough entirely left her without any apparent reaſon, as ſhe had long given up the uſe of internal medicines, and had made no change in her manner of living; but had truſted entirely to the eau de luce for occaſional relief.

XV. *Incontinence of Urine cured by the Uſe of the flexible Catheter. By* G. Mitchell, *Surgeon, at Wapping.* — The ſubject of this caſe was a woman, thirty-ſix years old, who, after being delivered of a large child by the aſſiſtance of the forceps, had a ſloughing of the vagina; in conſequence of which, a communication was formed between that and the urethra, very near the neck of the bladder. In this ſtate it was thought expedient that a flexible catheter ſhould be introduced into the urethra, and kept there conſtantly, if poſſible. The patient was inſtructed in the manner of managing it; and, we are told, that ſhe uſed it conſtantly about

 three three weeks, occaſionally removing it to eaſe herſelf, and to obſerve whether any water paſſed by the vagina. In a few days ſhe perceived that a leſs quantity was diſcharged that way than before; and at the end of the time above mentioned the complaint was entirely removed.

In a note to this paper the Society obſerve, that one of their members, (the late Dr. Hunter, if we miſtake not) who had ſeen many caſes of ſloughings in the urinary paſſages of women, was of opinion that, however proper and ſucceſsful the practice recommended by Mr. Mitchell may be, when the perforation happens to be in the urethra, or juſt at its beginning, there muſt be leſs expectation of a cure when the opening is in the body of the bladder. In this laſt caſe he feared the bladder would ſeldom or never bear the irritation of a catheter long enough to anſwer the purpoſe; and he particularly mentioned the caſe of one of his patients in this unhappy ſituation, who, though perfectly convinced of what importance the ſucceſs of the operation would be to her through life, was not able to bear the catheter in her bladder. The irritation never failed to bring on ſuch efforts as were were irrefiftible, till the catheter was driven down from the bladder into the urethra, and then the urine paffed involuntarily into the vagina; fo that the perforation remained fiftulous.

XVI. *A Letter to Dr. J. Fothergill on the Benefit of a refufcitated Salivation in the Cure of certain anomalous Symptoms, from Dr.* Matthew Dobfon.—The cafe related in this paper affords a ftriking confirmation of the efficacy of a practice adopted by Sir John Silvefter in three cafes defcribed in the third volume of the work now before us. — The patient was a young lady troubled with pimples in her face; for which fhe took fmall dofes of Plummer's pill till a flight falivation was excited. From the time the falivation ceafed fhe complained of more or lefs of pain about the region of the ftomach, difficulty of fwallowing, and other ftill more alarming fymptoms, fuch as convulfions, and frequent delirium. After opium and other anti-fpafmodics had been tried without affording her any relief, our author was induced to afcribe her complaints to the fudden fuppreffion of the falivation. Under this idea of the difeafe, the method of cure as fuggefted by Sir John Silvefter, was again to bring on the falivation. This was accordingly attempted, although the violence of the ſymptoms, and the extreme weakneſs of the patient, ſcarcely afforded the moſt diſtant hope that ſhe could ſurvive till the remedy had produced the deſired effect. Mercurial frictions were preſcribed, and calomel was at the ſame time given internally. In about four days a ſpitting came on, and the ſymptoms abated. The ptyaliſm continued for more than twenty days, and her complaints were entirely removed.— This hiſtory would, perhaps, have been more complete and ſatisfactory if the author had given us a farther account of the cutaneous eruption for which the mercurial courſe was originally preſcribed.

XVII. *The Hiſtory of an extraordinary Affection of the Brain, in a Letter to Dr. W. Hunter, from Dr.* John Smith, *Dr.* Martin Wall, *and Mr.* John Langford, *Surgeon.*—We have here a very curious account of a young gentleman, who was occaſionally ſubject to complaints in his head and bowels, which were at firſt aſcribed to worms: at length, ſymptoms intervened which ſeemed to indicate ſome morbid affection peculiar to the head itſelf, ſuch as, at times, a remarkable ſtupor, and a ſeeming temporary ſuſpenſion of all his intellectual faculties.

These

Thefe fymptoms were, at their laft attack, accompanied with an acute fever, which confined him to his bed, in a delirious ftate, for about a fortnight, when he died, at the age of twenty.

On diffection, the dura mater, pia mater, and fubftance of the brain juft within the fuperior tranfverfe ridge of the occiput, were found connected together by a bony concretion, broader than a fixpence, and thicker than a fhilling, which, on its edge and furfaces, was every where jagged and uneven, and which adhered alfo to the falx and longitudinal finus on the left fide.

After the death of the patient, our authors were informed that he formerly had received a violent blow on the hinder part of his head, and that for feveral years he had complained of a particular tendernefs and forenefs of that part of the head externally, (which correfponded with the feat of the internal concretion) efpecially when his hair was dreffed; and that the hair on that part was very thin. But in what manner the blow was given, what fymptoms he experienced, or what treatment he underwent at the time, they were unable to learn.

XVIII. *Obſervations on the Cure of Fluxes by ſmall Doſes of Ipecacuanha. By* J. Fothergill, *M. D.*—In this paper the author does not aim at deſcribing the different kinds of fluxes that occur in practice, in which ipecacuanha may be uſed with advantage; but confines his obſervations to what he terms an habitual diarrhœa depending on ſome irritating acrimony of the juices, accompanied with great weakneſs and irritability of the bowels. A complaint of this kind, we are told, is incident to perſons of both ſexes, and of different ages. In ſome it is accompanied with ſickneſs, bitter taſte, furred tongue, and ſome degree of fever: in others it occurs without any ſuch ſymptoms, weakening the patient merely by the frequency of the ſtools. In caſes of this ſort, where the viſcera were not injured in their ſtructure, our author has experienced good effects from the uſe of ipecacuanha in ſmall doſes. He directs a grain, a grain and a half, or two grains of this medicine to be taken, in bed, in a morning. At bed time a warm opiate, ſuch as the confect. Damocrat. or philonium, is to be adminiſtered in a doſe ſufficient to inſure an undiſturbed night. The ipecacuanha is to be repeated or omitted the next morning, according ing to its effects the preceding day. If its operation has been vehement, it is to be omitted till the morning following; but, at any rate, we are advifed to repeat the anodyne at bed time.

It moft commonly happens, we are told, that a very few dofes of thefe medicines, with proper attention to regimen, gradually reftrain thefe difcharges; and that the fame procefs, at longer intervals between the dofes of ipecacuanha, generally puts a ftop to them both fafely and effectually. And here the author takes occafion to obferve, that the ufual method of adminiftering fimilar dofes of this medicine, or of the vitrum antimonii ceratum, often does harm by exciting a conftant difpofition to purging, and, of courfe, not allowing fufficient time for the medicine to produce its due effect, and no more.

In refpect to regimen, Dr. Fothergill cautions us to enjoin a ftrict regard to quantity, and, at the fame time, advifes that the patient fhould confine himfelf, for a certain time, to one kind of food only. He thinks mutton as fuitable a diet as any other. He recommends the ufe of bark and chalybeates to eftablifh general health.

XIX. *The Case of a flatulent Tumor on the Head opened and cured. By Mr.* Lloyd, *Surgeon, at Wrexham.* — The tumor here defcribed was fituated on the juncture of the fagittal to the lambdoidal future, and, when our author firft faw it, was about the fize of a pigeon's egg. The patient was a middle-aged woman, who had always enjoyed good health; but about eight years previous to the appearance of the tumor, fhe had had a fall from a horfe on a pavement, which ftunned her fo much as to deprive her of her fenfes for fome minutes. She did not recollect, however, that any part of her head was at all bruifed, or otherwife hurt, then, or at any other time. The tumor, when preffed, gradually fubfided, and occafioned a noife in the left ear. In the courfe of about a twelvemonth it had increafed confiderably in bulk, and occafioned head-ach, dizzinefs, perpetual naufea, and a fenfation of numbnefs in her limbs, particularly in her arms. The tumor being now opened, was found to contain air only; but the cranium was carious the whole extent of the tumor, and the part it firft occupied much deftroyed, having the appearance of a honeycomb. Every fymptom difappeared on the opening of the tumor, and the wound wound healed in a ſhort time, without any ſenſible exfoliation.

XX. *Obſervations on the Gout. By* Alexander Small, *late Surgeon to the Ordnance in the Iſland of Minorca.* — In this paper, which was firſt publiſhed in French, in the *Journal de Medecine*, the author gives a very ſenſible and intereſting narrative of his own caſe, and of the good effects he has experienced from the uſe of emetic tartar and the Peruvian bark; for his account of which we muſt refer our readers to the paper itſelf.

XXI. *Dangerous Effects from eating a Quantity of ripe Berries of Belladonna; in a Letter from Mr.* Brumwell, *Surgeon to the Suſſex Militia.* — The effects produced by this poiſon, in four of the ſix ſoldiers whoſe caſes are here related, were a ſoreneſs and dryneſs of the throat, and a dilatation of the pupil of the eye. The other two, who ſwallowed a much larger quantity of the berries than their companions, had the ſame ſymptoms in a greater degree, and were likewiſe delirious. Active emetics and purgatives were adminiſtered, and they all recovered without any paralytic ſymptoms. One of the two, who were the moſt dangerouſly affected, continued delirious till the fourth day, when a

 ſecond

ſecond emetic was adminiſtered, which brought up a great quantity of the ſeeds; and after that he recovered very faſt.

The paper terminates with a letter from Mr. John Hoffmann, Chemiſt at Cambridge, in which he relates the effects of this poiſon in a dog; and likewiſe mentions the remarkable ſymptoms, reſembling hydrophobia, excited in a greyhound that ſwallowed two drachms of camphor.

XXII. *Caſe of a Feather or Pen, twelve Inches in Length, which was fortunately extracted from the Oeſophagus of a Man who had put it into his Throat to excite vomiting, and had let it ſlip down. By Mr.* Samuel Croker King, *Surgeon, at Dublin.*— This caſe is nearly ſimilar to that related by Dr. Cleghorne in the third volume of the work we are reviewing.

XXIII. *Caſe of a diſeaſed Kidney. By* George Pearſon, *M. D. of Doncaſter.*—The ſubject of this caſe was a boy, four years and a half old, who, after being for ſeveral months occaſionally troubled with pain of the belly, eſpecially in the lower part, began to have a ſwelling of the abdomen, which gradually increaſed to an enormous ſize, and was extremely tenſe; but the right ſide of it was larger and harder than the left.

left. The quantity of his urine diminiſhed, and an obſcure fluctuation being felt in the lower part of the abdomen, a puncture was made with a trocar, but without evacuating any watery fluid. The limits of our work will not allow us to follow the author through the whole of the accurate account he gives of the ſymptoms and progreſs of the diſeaſe till the death of the patient, or to mention the various remedies that were adminiſtered for his relief. We ſhall, therefore, content ourſelves with obſerving, that, at times, when free from the pain of his belly, he was able to walk abroad, ſlept well, and was cheerful and, in all reſpects, eaſy, but for the weight of the abdomen. His pulſe varied from 110 to 140 ſtrokes in a minute. In the latter ſtages of the diſeaſe he complained much of pain in his arms and neck. The veins of the abdomen and thorax were exceedingly enlarged; his legs and thighs were oedematous; and, at length, he was ſo much emaciated as to be unable to get out of bed. He died in September, 1778, twenty months from the firſt appearance of the diſeaſe. On diſſection, the right kidney was found in a diſeaſed ſtate, and enlarged to an enormous ſize, weighing ſixteen pounds and ten ounces. The left kidney, and the other contents of the abdomen, were in a natural ſtate.

Dr. Pearſon adds two letters from Dr. Alexander Monro and Dr. Charles Webſter, giving accounts of a ſimilar caſe that occurred about the year 1776, in the Infirmary at Edinburgh. The patient mentioned by theſe gentlemen was a woman, aged fifty, who complained of nothing but a gradual diſtention of her abdomen, till it attained an enormous ſize. As an obſcure fluctuation was felt, ſhe was tapped twice; but the diſcharge was inconſiderable. On diſſection, the left kidney was found to be exceedingly indurated, and to weigh forty-five pounds.—Dr. Monro, in his letter to our author, obſerves, that, "from the ſoftneſs of "tumors containing water; the ſmoothneſs of "the whole, or of the parts which compoſe "them; their indolence, and fluctuation felt "when they are ſtruck, he apprehends they "may, with little or no exception, be diſtin-"guiſhed from other caſes."

Sauvages, in his Noſologia Methodica, has collected ſome inſtances of this diſeaſe, from authors, under the name of *Phyſconia Renalis.*

XXIV. *An Inſtance of the good Effects of Opium in a dangerous Caſe of Retention of Urine. By* John Pearſon, *Surgeon to the Lock Hoſpital, and to the Public Diſpenſary in Carey Street.*—The retention of urine in this caſe was the effect of a gonorrhœa; and the ſtrictures and inflamed ſtate of the urethra rendered the introduction of a catheter impracticable. Warm bathing, clyſters, &c. were tried without any good effect. At length, our author determined to adminiſter opium liberally, with a view, as he expreſſes it, to 'ſuſpend the tonic action of the moving 'fibres,' hoping thereby to deprive the ſphincter of the bladder of its contractile power. The patient took a grain of *extr. theb.* every four hours, and, after the fourth doſe, fell aſleep; while he was aſleep, his urine flowed from him involuntarily, and in great quantity. After ſleeping ſix hours, he awoke, very much relieved; and, from that period, his inflammatory ſymptoms gradually diſappeared.

XXV. *On Cataracts. By Mr.* James Lucas, *Surgeon to the Leeds Infirmary.* — The remarks contained in this paper are evidently the production of an experienced and judicious writer. In the cure of a cataract, he inclines moſt in favour of depreſſion. In couching, he prefers a round a round needle to the flat one commonly ufed. For this improvement, which is now generally adopted by the furgeons of the Infirmary at Leeds, he acknowledges himfelf indebted to Baron Hilmer, an itinerant oculift. Mr. Lucas obferves alfo, that the needles in general ufe are heavier and longer, both in the handle and needle, than is neceffary. That which he employs does not weigh half a drachm, and is only about four inches and a half in length: the point of it is a little flattened, and the handle has a little flatnefs correfponding with the point.

Several cafes are related in the courfe of the paper, and, among others, thofe of the five children of the Rev. Mr. Hall, of Leaven, near Beverley, who were all born blind. They are faid to be, in different degrees, idiots, and are in almoft perpetual motion with one limb or other, in a manner refembling St. Vitus's dance; fo that there is probably fome peculiarity in the ftructure of their brain.

XXVI. *On the Uncertainty of the Signs of Murder in the Cafe of Baftard Children. By the late* William Hunter, *M. D. F. R. S. &c.* — What is commonly underftood to be the murder of a baftard child by the mother, would, if the real circumcircumſtances were fully known, be allowed to be a very different crime in different circumſtances. The excellent paper now before us is intended to point out theſe circumſtances, and to remove the prejudices which have too generally and too fatally prevailed on this ſubject.— "I have ſeen," ſays the benevolent author, "the private as well as the public virtues, the "private as well as the more public frailties of "women, in all ranks of life. I have been a "witneſs to their private conduct, when they "were preparing themſelves to meet danger; "and have heard their laſt and moſt ſerious "reflections, when they were certain they had "but a few hours to live.—This knowledge of "women has enabled me to ſay, though, no "doubt, there will be many exceptions to the "general rule, that women who are pregnant "without daring to avow their ſituation, are "commonly objects of the greateſt compaſ-"ſion, and generally are leſs criminal than the "world imagine."—In ſome, he obſerves, (and it is to be hoped, *rare)* inſtances, the murder of a baſtard child by the mother is a crime of the deepeſt dye; but he is perſuaded that the greateſt number of what are called murders of baſtard children, are of a very different ferent kind. The mother, having an unconquerable ſenſe of ſhame, and anxious to preſerve her character, has not the reſolution to meet and avow infamy. In proportion as ſhe every day ſees the danger of a diſcovery greater and nearer, her mind becomes overwhelmed with terror and deſpair. In this ſituation, many of theſe women, who are afterwards accuſed of murder, would deſtroy themſelves, if they did not know that ſuch an action would infallibly lead to an inquiry, which would proclaim what they are ſo anxious to conceal. In this perplexity, they are meditating different ſchemes for concealing the birth of the child; but are wavering between difficulties on all ſides, putting the evil hour off, and truſting too much to chance and fortune. In that ſtate, often they are overtaken ſooner than they expected; their ſchemes are fruſtrated; their diſtreſs of body and mind deprives them of all judgment and rational conduct; they are delivered by themſelves wherever they happen to retire in their fright and confuſion; ſometimes dying in the agonies of child-birth, and ſometimes, being quite exhauſted, they faint away, and become inſenſible of what is paſſing; and when they recover a little ſtrength, find that the the child, whether ftill born or not, is completely lifelefs.—This is a part, a fmall part only, of the author's reafoning on the fubject; and he illuftrates his obfervations by appofite cafes.

Having fhewn that the fecreting of the child amounts, at moft, to fufpicion only, Dr. Hunter proceeds to the moft important queftion of all, viz. If, in the cafe of a concealed birth, it be clearly made out that the child had breathed, may we infer that it was murdered? and he brings feveral convincing arguments to prove that we cannot; and that it is certainly a circumftance, like the laft, which amounts only to fufpicion.

XXVII. *Three Cafes of Mal-Conformation in the Heart* *. *By the late* William Hunter, *M. D. F. R. S.* &c.— Thefe cafes were intended to accompany one of a fimilar kind communicated to the Society by Dr. Pulteney, and fince publifhed in the third volume of Medical Tranfactions. The fubject of the firft of Dr. Hunter's cafes was a male child, who, from the moment of its birth, was obferved to have a very laborious refpiration. Its fkin was every where black; but the moft uncommon appearance was the motion of its heart, which was

* See page 407.

ſo violent, that it could be ſeen outwardly from a conſiderable diſtance. The child died at the end of thirteen days; and, on diſſection, the great peculiarity of the caſe was found to be a preternatural conformation of the pulmonary artery, which, at its beginning from the right ventricle, was contracted into a ſolid ſubſtance, ſo as to be abſolutely impervious. The right ventricle had hardly any cavity left; and it was evident that the returning blood of both *cavæ* had paſſed through the foramen ovale and left ventricle into the aorta; from which, by the *canalis arterioſus*, a ſmall portion had been conveyed, by a retrograde motion, into the left branch of the pulmonary artery; and it was this ſmall portion of the blood only which had received the benefit of reſpiration.

The ſecond caſe is that of a young gentleman who lived till he was thirteen. Dr. Hunter firſt ſaw him when he was eight years old. He was as tall as might be expected at his age, but was remarkably thin. This thinneſs, however, appeared to be his natural habit, and not the effect of conſumption. His complexion had been always dark or tending to black. His moſt diſtreſſing ſymptoms were fits, on the approach of which he grew oppreſſed at his heart, became faint, grew dark in his colour, and, at laft, almoft black; fell down and feemed infenfible. He commonly foon came out of the fit, with fobbing and yawning, and a fenfe of fatigue. Any hurry of mind, or brifk motion of his body, would generally occafion a fit. But fome years before his death, he had found out, by his own obfervation, that, when the fit was coming on him, he could efcape it altogether, or, at leaft, confiderably leffen its duration and violence, by inftantly lying down upon the carpet, on his left fide, and remaining immoveable in that pofition for about ten minutes.

Dr. Hunter gave it as his opinion, that in this cafe there was a peculiarity of conftruction about the heart; and this conjecture was confirmed, after the death of the patient, by diffection. The pulmonary artery, at its origin from the right ventricle, was fo fmall, that it would barely give paffage to a fmall probe. Another peculiarity was alfo difcovered in the ftructure of the heart itfelf; the *feptum cordis* being deficient, or perforated at the bafis of the heart.

To thefe two cafes, which are illuftrated by engravings, Dr. Hunter has added a third,

 fomefomewhat fimilar, from his Anatomical Mufeum. Of the hiftory of the child, whofe heart is the fubject of this account, nothing more is known than that it was ftill born at the end of fix months. On examining its heart, a hole was found in the feptum, at its bafis, large enough to allow a goofe quill to pafs with eafe from either ventricle to the other. This hole, from the fmoothnefs of its edge, plainly appeared to have exifted from the firft formation.

In fome very interefting reflections annexed to the preceding cafes, Dr. Hunter, among other things, obferves, that the dark and gray complexion of the fubjects of the firft and fecond cafes correfponds with the obfervations of philofophers, that the blood takes it bright hue in the lungs from refpiration. — In the fecond cafe, why there was not the full effect of nutrition, or why he did not increafe in thicknefs as well as in length, he leaves to be explained by thofe who know the whole procefs of nutrition, and can tell us why blood does not anfwer the purpofe fo well that has not all paffed through the lungs.

In preternatural cafes, he obferves, we often find that what is deficient is compenfated in fome other way. Thus, in the fecond cafe, where the pulmonary artery was preternaturally ſmall, there was a communication, through the ſeptum, between the right ventricle and the aorta, as if formed on purpoſe to ſupply that defect. In very early life, he remarks, thouſands of animal ſyſtems may be loſt by reaſon of ſome preternatural ſtructure, without ſuch compenſation being provided as to allow the fabric to go on; and hence he is led to conjecture that miſcarriages at different periods of pregnancy may be owing to uncompenſated defect, which allows the fœtus to live only ſo long, and no longer.

XXVIII. *The ſucceſsful Cure of a ſevere Diſorder of the Stomach by Milk taken in ſmall Quantities at once. By the late* William Hunter, *M. D. F. R. S.* &c.—The ſubject of this caſe, which the author uſed to mention in his lectures, was a young gentleman about eight years old, who had long laboured under great pain in his ſtomach, frequent and violent vomitings, great weakneſs, and ſo great a waſting of fleſh, that, Dr. Hunter ſays, he hardly ever ſaw a human creature more emaciated. When he firſt ſaw the patient, the diſorder was of ſome months ſtanding, and had been daily growing more deſperate. A variety of medicines, and, among others, others, opium, had been tried without the leaſt good effect. Opium, at firſt, afforded a temporary relief; but, after a little uſe, that likewiſe was ſoon brought up again by vomiting. It was in this hopeleſs ſtate of the caſe that our author was conſulted. As nothing could with certainty be determined concerning the cauſe of the complaint, he contented himſelf with recommending friction with warm oil and a warm hand, before a fire, over and all around the patient's ſtomach, every morning and evening; and at the ſame time cautioning the patient's friends carefully to avoid offending his weak ſtomach either with the quantity or quality of what might be taken down; as milk ſeemed to be the propereſt nouriſhment, a trial of it, beginning with a ſpoonful, or even a ſmaller quantity, was adviſed. "If he can but "bear the ſmalleſt quantity," ſaid the Doctor to the patient's father, "you will be ſure "of being able to give him nouriſhment. "Let it be the ſole buſineſs of one perſon to "feed him. If you ſucceed in the beginning, "perſevere with great caution, and proceed "very gradually to a greater quantity, and to "other fluid food, eſpecially to what his own "fancy may invite him."

Dr. Hunter heard nothing of the cafe till after two or three months, when the patient's father came to him with a joyful countenance, and informed him that the plan had been purfued with fcrupulous exactnefs, and with aftonifhing fuccefs; that his fon had never vomited fince his vifit to him, and that he was daily gaining flefh and ftrength. In a word, we are told, that he recovered completely, and grew to be a healthy and very ftrong man.

This cafe was communicated to the Society at the requeft of Mr. William Hey, Surgeon at Leeds, who, in a letter to Dr. Hunter, which is given in the form of an appendix, relates four inftances of the fuccefs of a fimilar plan of diet in cafes of obftinate vomiting. The recollection of the cafe he had heard related by Dr. Hunter in his lectures, firft induced him to adopt this method.

XXIX. *Cafe of Recovery from apparent Death, in confequence of taking a large Dofe of Opium. By* Thomas Whately, *Surgeon, Old Jewry.* — We have here a very interefting account of the cafe of a fervant of a druggift at Derby, who fwallowed about half an ounce of folid opium. At the end of half an hour, when Mr. Whately firft faw him, he was fometimes in a kind of delirium delirium and unable to ftand, and at other times was difpofed to fleep. Large and repeated dofes of emetic tartar were adminiftered, and, after feveral vomitings, his complaints were fo much abated, that, at the end of about three hours and a half, he was able to walk about, and was apparently more cheerful; but having at this time been left alone for two or three minutes, he was found in a ftate of apparent death. His face was pale, cold, and cadaverous; his lips were of a blackifh white; his eyes had loft all luftre; his eyelids remained in any pofture they were placed in; and his refpiration feemed totally gone; but he had ftill a fmall irregular pulfe. A pair of common bellows were now applied to his mouth, while a handkerchief was held round his mouth and noftrils. In this manner refpiration was continued for feveral minutes, without any apparent effect; but being unwilling to relinquifh the cafe, Mr. Whately renewed the operation, and, at the end of feven minutes, a kind of weak croaking was perceived at the time the air came out in expiration. At the next expiration it was rather ftronger, and gradually increafed till there were more evident figns of life, when his natural countenance returned, and he knew thofe who were about him, and was able to anfwer rationally. Still, however, he was remarkably fleepy, and, unlefs attended to, would in a moment be gone; but by repeating the emetic tartar, (of which he took upwards of forty grains at different times in the courfe of twelve hours) and fupplying him plentifully with warm water, he was fo well recovered the next morning as to require no farther attendance. The relief that was obtained at each evacuation was owing to the partial removal of the caufe; for as no folid opium could be obferved in the evacuated liquor, that part only was difcharged which was diffolved. From this circumftance, Mr. Whately is led to infer, that a much longer attention is required to a cafe where folid opium is fwallowed than when it is taken in a liquid form.

XXX. *A Sketch of the epidemic Difeafe which appeared in London towards the End of the Year* 1775. *By the late* John Fothergill, *M. D.*

XXXI. *An Account of a fatal Difeafe of the Stomach, by* Michael Morris, *M. D. F. R. S.; with a Relation of the Appearances on opening the Body, by Mr.* Henry Watfon, *F. R. S. and Surgeon to the Weftminfter Hofpital.*—The difeafe here defcribed was found to have been occa-

 fioned fioned by a clufter of tubercles within the pylorus, which almoft totally obftructed the paffage from the ftomach into the duodenum. An engraving is added of the appearances on diffection, which are defcribed with great accuracy by Mr. Watfon.

